# Effect of low-dose atropine eyedrops on pupil metrics: results after half a year of treatment and cessation

**DOI:** 10.1007/s00417-022-05863-8

**Published:** 2022-11-19

**Authors:** Wei-Ling Bai, Jia-He Gan, Shifei Wei, Shi-Ming Li, Wen-Zai An, Xin-Tong Liang, Jia-Xin Tian, Lei Yin, Ningli Wang

**Affiliations:** 1 Beijing Institute of Ophthalmology, Beijing Tongren Eye Center, Beijing Tongren Hospital, Capital Medical University, Beijing Ophthalmology & Visual Sciences Key Laboratory, Beijing, China; 2 Department of Ophthalmology, School of Medicine, Shanghai East Hospital, Tongji University, No. 150 Jimo Road, Pudong New District, 200120 Shanghai, China; 3 Department of Ophthalmology, Zhengzhou First People’s Hospital, Zhengzhou, Henan China

**Keywords:** Myopia, Low-dose atropine, Pupil, Children

## Abstract

**Purpose:**

To evaluate the effect of low-dose atropine eyedrops on pupil metrics.

**Methods:**

This study was based on a randomized, double-masked, placebo-controlled, and cross-over trial in mainland China. In phase 1, subjects received 0.01% atropine or placebo once nightly. After 1 year, the atropine group switched to placebo (atropine-placebo group), and the placebo group switched to atropine (placebo-atropine group). Ocular parameters were measured at the crossover time point (at the 12th month) and the 18th month.

**Results:**

Of 105 subjects who completed the study, 48 and 57 children were allocated into the atropine-placebo and placebo-atropine groups, respectively. After cessation, the photopic pupil diameter (PD) and mesopic PD both decreased (− 0.46 ± 0.47 mm, *P* < 0.001; − 0.30 ± 0.74 mm, *P* = 0.008), and the constriction ratio (CR, %) increased (4.39 ± 7.54, *P* < 0.001) compared with values at the crossover time point of the atropine-placebo group; pupil metrics of the atropine-placebo group had no difference from the values at the crossover time point of the placebo-atropine group. After 6 months of treatment, the photopic PD and the mesopic PD increased (0.54 ± 0.67 mm, *P* < 0.001; 0.53 ± 0.89 mm, *P* < 0.001), the CR (%) decreased (− 2.53 ± 8.64, *P* < 0.001) compared with values at the crossover time point of the placebo-atropine group. There was no significant relationship between pupil metrics and myopia progression during 0.01% atropine treatment.

**Conclusion:**

Pupil metrics and the CR could return to pre-atropine levels after cessation. Pupil metrics had no significant effect on myopia progression during treatment.


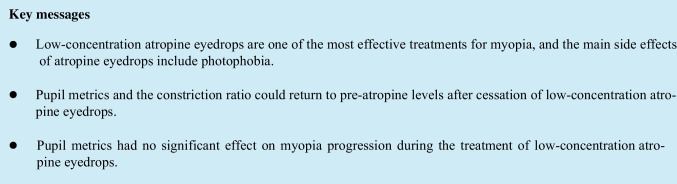


## Introduction

The increasing prevalence of myopia and high myopia has brought significant economic and social burdens [1, 2]. At present, clinical guidelines for myopia control include orthokeratology lenses, contact lenses with peripheral defocus design, maximizing time spent outdoors, and low-concentration atropine eyedrops [3–8]. Studies have shown that low-concentration atropine eyedrops are one of the most effective treatments for myopia [8–10]. However, the ideal atropine concentration has yet to be determined since the higher the concentration of atropine eyedrops, the better the effect of controlling myopia development, but with more apparent adverse effects and more obvious rebound after drug cessation [11–13].

The main side effects of atropine eyedrops are photophobia, glare, and near blur [9, 14, 15]. The ocular symptoms may be related to mydriasis and impaired pupillary light reflex (PLR) [16]. Differences in atropine concentration, race, and follow-up durations among studies may have contributed to different proportions of photophobic glare and near-blurred vision [17]. There were relatively limited studies reporting changes in pupil metrics after the use of atropine [12, 18, 19]. Fu et al. reported that 0.02% and 0.01% atropine increased pupil diameter (PD) similarly after 4 months (0.87 mm) and 12 months (0.77 mm; *P* = 0.55) of treatment [19]. Yam and colleagues showed that the increase in pupil size followed a concentration-related response [20]. In addition, Chen et al. suggested that a larger PD induced a higher intensity of myopic shift in the peripheral retina, exerting a more significant suppressive effect on axial growth [21]. As the assessment of pupil appearance and PLR may inform us of the integrity of the autonomic nervous system, and measures of pupillary metrics are safe and noninvasive to characterize the mechanism of drug action, it is necessary to monitor changes in pupil metrics during atropine treatment and after cessation [22, 23].

In this study, children who had used 0.01% atropine eyedrops for 1 year were followed up for another 6 months after drug cessation. In addition, we also evaluated the change of pupil metrics of children using 0.01% atropine during the same period and explored whether pupil metrics played a role in controlling myopia progression during atropine treatment.

## Methods

### Study design and setting

This study was based on a randomized, double-masked, placebo-controlled, and cross-over trial which comprised 2 phases in mainland China. The detailed design and methods have been described previously in phase 1 [8]. Briefly, children aged 6 to 12 years old with spherical equivalent (SE) refraction range of − 1.00 to − 6.00 D in both eyes, astigmatism of less than 1.50 D in both eyes, and intraocular pressure of less than 21 mmHg were enrolled in this study. In phase 1 (the first year), 220 subjects were randomized to receive either 0.01% placebo or atropine eye drops at bedtime every night in both eyes for 1 year. In phase 2 (the second year), the placebo group was crossed over to the 0.01% atropine group (referred to as the “placebo-atropine group”), and the 0.01% atropine group was crossed over to the placebo group (referred to as the “atropine-placebo group”) for 1 year. All eye drops were prepared in mono-dose preparation by Shenyang Xingqi Pharmaceutical Co, Ltd (Shenyang, PR. China). Our study reported the results from the crossover time point (at the 12th month) to the 18th month.

The study adhered to the tenets of the Declaration of Helsinki and was approved by the Ethics Committee of Beijing Tongren Hospital. All participants provided written informed consent after agreeing to enrollment. The trial was registered on the Chinese Clinical Trial Registry (http://www.chictr.org.cn/index.aspx). The registration number is ChiCTR-IOR-17013898 [24].

### Outcome measurements

From the crossover time point (at the 12th month) to the 18th month, subjects underwent the same standardized ophthalmic examinations as in phase 1. Measurements were taken from 9:00 to 12:00 at the weekend. The measurement of pupil sizes was examined before measuring the axial length (AL) and refractive error. The OPD-Scan III (Nidek, Japan) was applied to measure mesopic and photopic pupil sizes. The protocol parameters of the device were reset before each measurement. We had patients sit directly across from the examiner; participants were asked to fixate on a distant object to relax their accommodation with the left eye that was not being measured. Then, the mesopic (background intensity was 0 μw) pupil size was measured three times and averaged using the OPD-Scan III, followed by photopic illuminance (background intensity as 50 μw). For each set of measurements, the average value of the first three consecutive data captures with differences less than 0.50 mm was used for analyses. We assessed the following parameters using the following equations [25]:

$$\mathrm{PLR}\:=\:\mathrm{mesopic}\;\mathrm{PD}-\mathrm{photopic}\;\mathrm{PD}$$

$$\mathrm{Constriction}\;\mathrm{ratio}\:=\:(\mathrm{mesopic}\;\mathrm{PD}-\mathrm{photopic}\;\mathrm{PD})/\mathrm{mesopic}\;\mathrm{PD}\:\times\:100\%$$

### Statistical analysis

Statistical analyses were performed using commercial software (SPSS version 25.0; SPSS, Inc., Chicago, IL, US). Mean values for ocular parameters were calculated from the right eyes. Categorical data were represented as counts (frequencies). Mean ± standard deviation values were used to describe continuous variables. The Kolmogorov–Smirnov test was used to examine the distributions of continuous data. Continuous data with normal distributions were analyzed with paired *T*-tests within the group. Continuous variables with abnormal distributions were analyzed with Mann–Whitney U-tests or Wilcoxon rank-sum tests. The chi-square test was used to assess the difference in gender between the two groups. The change of parameters was defined by the difference between the crossover time point (at the 12th month) and the corresponding follow-up values.

To explore whether the pupil metrics can recover after cessation, an independent-sample *T*-test was used to compare pupillary parameters of the atropine-placebo group (values at the 18th month) with the placebo-atropine group (values at the 12th month).

The multivariable regression model was conducted to investigate whether pupil metrics contributed to myopia progression during atropine treatment. Univariable analysis was also performed to assess the associated factors for myopia progression. Multivariable analysis was performed using variables with *P* values less than 0.2 in univariable analysis. A *P* value < 0.05 with two-sided was considered statistically significant.

## Results

In this study, one hundred and five (47.73%) children with available data were enrolled. Forty-eight subjects were in the atropine-placebo group and fifty-seven were in the placebo-atropine group. No significant difference was found between the demographic characteristics of the atropine-placebo group and the placebo-atropine group at the crossover time point (Table 1).

**Table 1:** Demographics and characteristics at crossover time points in the atropine-placebo group and placebo-atropine group (mean ± SD)

	Atropine-placebo group (*N* = 48)	Placebo-atropine group (*N* = 57)	*P* value
Variables	Mean ± SD	Mean ± SD	
Age (yrs)	10.92 ± 1.70	11.08 ± 1.55	0.62
Sex (male, %)	21 (43.75%)	32 (56.14%)	0.21
Intraocular pressure (mmHg)	16.70 ± 3.45	14.69 ± 4.29	0.10
Cycloplegic spherical equivalent (D)	− 3.14 ± 1.32	− 3.42 ± 1.44	0.31
Age at myopia onset (yrs)	7.96 ± 1.50	8.22 ± 1.67	0.41
Near work, (h/d)	3.27 ± 1.11	3.31 ± 1.34	0.87
Time outdoors, (h/d)	1.35 ± 0.57	1.60 ± 0.62	0.35

For the atropine-placebo group, 6 months after treatment cessation, the photopic PD and mesopic PD decreased significantly compared with the end of atropine treatment (3.86 ± 0.55 mm vs. 3.40 ± 0.42 mm, *P* < 0.001; 5.91 ± 0.58 mm vs. 5.61 ± 0.65 mm, *P* = 0.008); the constriction ratio (%) increased significantly (34.73 ± 5.45 mm vs. 39.12 ± 6.58 mm, *P* < 0.001; Tables 2, 3). For the placebo-atropine group, 6 months after atropine treatment, the increase of photopic PD and mesopic PD was significant (3.36 ± 0.46 mm vs. 3.90 ± 0.61 mm, *P* < 0.001; 5.50 ± 0.75 mm vs. 6.04 ± 0.65 mm, *P* < 0.001) and the constriction ratio decreased (38.67 ± 6.34 vs. 36.14 ± 6.62, *P* = 0.03) compared to pre-atropine screening (at the 12th month). The PLR of atropine-placebo group and placebo-atropine group changed slightly (2.05 ± 0.34 mm vs. 2.21 ± 0.51 mm, *P* = 0.07; 2.15 ± 0.53 mm vs. 2.17 ± 0.47 mm, *P* = 0.81; Tables 2, 3).

**Table 2:** Biometric parameters during the follow-up period (mean ± SD)

Time	Atropine-placebo group					Placebo-atropine group			
	Photopic pupil diameter (mm)	Mesopic pupil diameter (mm)	Pupillary light reflex (mm)	Constriction ratio (%)		Photopic pupil diameter (mm)	Mesopic pupil diameter (mm)	Pupillary light reflex (mm)	Constriction ratio (%)
Crossover	3.86 ± 0.55	5.91 ± 0.58	2.05 ± 0.34	34.73 ± 5.45		3.36 ± 0.46	5.50 ± 0.75	2.15 ± 0.53	38.67 ± 6.34
6 months	3.40 ± 0.42	5.61 ± 0.65	2.21 ± 0.51	39.12 ± 6.58		3.90 ± 0.61	6.04 ± 0.65	2.17 ± 0.47	36.14 ± 6.62
*t*	− 6.78	− 2.79	1.87	4.03		6.10	4.55	0.25	− 2.21
*P*	< 0.001	0.008	0.07	< 0.001		< 0.001	< 0.001	0.81	0.03

**Table 3:** Comparison of mean change in pupil metrics of two groups after 6 months (mean ± SD)

	Photopic pupil diameter (mm)	Mesopic pupil diameter (mm)	Pupillary light reflex (mm)	Constriction ratio (%)
Atropine-placebo group	− 0.46 ± 0.47	− 0.30 ± 0.74	0.16 ± 0.61	4.39 ± 7.54
Placebo-atropine group	0.54 ± 0.67	0.53 ± 0.89	0.02 ± 0.69	− 2.53 ± 8.64
*t*	8.71	− 5.16	1.10	4.33
*P*	< 0.001	< 0.001	0.27	< 0.001

The change in pupil metrics, including photopic PD, mesopic PD, and constriction ratio, differed between the two groups (*P* < 0.001), while the change in PLR showed no significant difference (*P* = 0.27, Table 3). Six months after cessation, pupil metrics of the atropine-placebo group had no difference from the values of the placebo-atropine group at the 12th month (Table 4).

**Table 4:** Comparison of the change in pupil metrics of the atropine-placebo group (6 months after cessation) and placebo-atropine (at cessation time point) in photopic pupil diameter (mean ± SD)

	Photopic pupil diameter (mm)	Mesopic pupil diameter (mm)	Pupillary light reflex (mm)	Constriction ratio (%)
Atropine-placebo group	3.40 ± 0.42	5.61 ± 0.65	2.21 ± 0.51	39.12 ± 6.58
Placebo-atropine group	3.36 ± 0.46	5.50 ± 0.75	2.15 ± 0.53	38.67 ± 6.34
*t*	1.00	1.01	0.50	0.02
*P*	0.32	0.32	0.62	0.98

**Table 5:** Univariable analysis and multivariable analysis of associations between myopia progression and ocular parameters in placebo-atropine group

Parameters	Change of cycloplegic spherical equivalent							Change of axial length					
	Univariable			Multivariable					univariable			Multivariable	
	*β*	*P* value		*β*	*P* value				*β*	*P* value		*β*	*P* value
General parameters													
Age (yrs)	0.15	0.29							− 0.085	0.54			
Sex (male, %)	0.16	0.22							0.075	0.57			
Age at myopia onset (yrs)	0.19	0.18		0.09	0.57				− 0.06	0.66			
Near work, (h/d)	0.21	0.13		0.12	0.43				0.23	0.05		0.31	0.034
Time outdoors, (h/d)	0.38	0.01		0.32	0.05				0.075	0.63			
Ocular parameters													
Intraocular pressure (mmHg)	− 0.048	0.74							0.050	0.73			
Cycloplegic spherical equivalent (D)	0.095	0.50							0.0035	0.98			
Axial length (mm)	0.15	0.31							− 0.16	0.26			
Photopic pupil diameter (mm)	0.10	0.45							0.04	0.77			
Mesopic pupil diameter (mm)	− 0.12	0.38							0.11	0.47			
Pupil light reflex (mm)	− 0.17	0.21							0.099	0.47			
Constriction ratio (%)	− 0.21	0.13		− 0.0036	0.99				0.064	0.641			
Change of photopic pupil diameter (mm)	− 0.20	0.16		− 0.17	0.45				0.18	0.199		0.18	0.32
Change of mesopic pupil diameter (mm)	− 0.13	0.34							0.07	0.66			
Change of pupil light reflex (mm)	0.0050	0.97							− 0.11	0.44			
Change of constriction ratio (%)	0.093	0.51							− 0.23	0.09		− 0.10	0.57

Pupil light reflex, calculated as Mesopic pupil diameter - Photopic pupil diameter; Constriction ratio, calculated as (Mesopic pupil diameter - Photopic pupil diameter) / Mesopic pupil diameter ×100%

Table 5 shows the association between myopia progression and pupil metrics using univariable analysis. The univariable analysis showed that the change of SE was related to age at myopia onset (*P* < 0.2; *β*, 0.19), time spent on near work (*P* < 0.2; *β*, 0.21), time outdoors (*P* < 0.05; *β*, 0.38), constriction ratio at the crossover time point (*P* < 0.02; *β*, − 0.21), change of photopic PD (*P* < 0.2; *β*, − 0.20). To eliminate nonsignificant factors, we conducted multivariable linear regression analysis. The change of SE was no longer associated with age at myopia onset (*P* = 0.57; *β*, 0.09), time spent on near work (*P* = 0.43; *β*, 0.12), time outdoors (*P* = 0.05; *β*, 0.32), constriction ratio at the crossover time point (*P* = 0.99; β, − 0.0036) or change of photopic PD (*P* = 0.45; *β*, − 0.17, Table 5).

The univariable analysis showed that the change of AL was related to time spent on near work (*P* < 0.2; *β*, 0.23), change of photopic PD (*P* < 0.2; *β*, 0.20), change of CR (%) (*P* < 0.2; *β*, − 0.23). We conducted multivariable linear regression analysis to eliminate nonsignificant factors. The change of photopic PD (*P* = 0.32; *β*, 0.18) and change of CR (%) (*P* = 0.57; *β*, − 0.10) was no longer associated with the change of AL (Table 5); while, the time spent on near work (*P* < 0.05; *β*, 0.31) was still related to the change of AL.

## Discussion

This study found that pupil size and the constriction ratio could return to pre-atropine levels after cessation. A once-nightly dose of 0.01% atropine eyedrops induced the PD increase and decreased constriction ratio but did not influence the PLR. The condition of pupil metrics before atropine treatment and the changes of pupillary parameters during treatment had no significant effect on myopia progression.

As a nonselective muscarinic antagonist agent, low-dose atropine for controlling myopia progression has aroused general interest, and its efficacy has been preliminarily recognized in recent years [12]. The eyedrops block both the pupillary sphincter and ciliary muscle, causing its main ocular symptom of photophobia [26, 27]. In clinical trials of different concentrations of atropine, some subjects dropped out due to the side effect [28–30].

Studies have reported that children in 0.01% and 0.02% atropine groups were photophobic in bright sunlight at the beginning of the treatment, but the symptom was not obvious in normal indoor lighting or when the sunlight was not intense outside [31]. Most subjects could adjust to photophobia caused by slightly dilated pupils after a period of atropine treatment [31].

According to Yam’s report, after 4 months of 0.01% atropine eyedrops treatment, photopic PD increased by 0.26 ± 0.83 mm and mesopic PD increased by 0.18 ± 0.46 mm; after 8 months of treatment, photopic PD increased by 0.41 ± 0.80 mm and mesopic PD increased by 0.16 ± 0.46 mm [10]. In our study, the increase of photopic and mesopic PD was 0.54 ± 0.67 mm and 0.53 ± 0.89 mm. It should be noted that the increase in PD in our study was larger than that in Yam’s, which may be related to the different periods. Another study that used 0.01% atropine eyedrops found that photopic PD increased by 0.77 mm after the 4-month treatment and 0.74 mm after the 8-month treatment (pupil measurement was under 300 to 310 lx illumination) [19]. The variation was a bit larger than ours. The difference may be related to the mode of the instrument measurement and the background light intensity set. And the effect of atropine varies with race-related melanin levels within the iris [16].

Most studies reported the psychophysical changes 1 year after the instillation of the drops. In a meta-analysis, 1-year randomized controlled trials showed both photopic (weighted mean difference, 0.35 mm; 95% CI = 0.02, 0.68) and mesopic PD (weighted mean difference, 0.51 mm; 95% CI = 0.31, 0.71) increased significantly in the 0.01% atropine group compared with control groups [11]. As previous studies reported, subjects using 0.01% atropine for 1 year showed an increase in photopic PD (ranging from 0.26 to 1.2 mm), and mesopic PD (ranging from 0.09 to 1.15 mm) (Table 6) [9, 10, 19, 32, 33]. Cooper et al. proposed that when pupil dilation exceeds 3 mm, noticeable photophobia could appear in daily visual tasks [16]. In Cooper’s clinical trial, among the 0.025%, 0.05%, 0.08%, 0.125%, 0.166%, 0.225%, 0.333%, 0.40%, and 0.50% atropine, 0.02% atropine was the highest concentration which did not result in clinical symptoms [16]. The pupil dilation of our placebo-atropine group was below the threshold of no more than 3 mm.

**Table 6:** Summary of design and pupillary parameters from RCTs case–control studies that include 0.01% atropine

Source	Design	Country	Duration			Device	Treatments	Mean (SD)		
			Age range, y	Size	Duration (*y*)			Baseline age, *y*	Change of photopic pupil diameter, mm	Change of mesopic pupil diameter, mm
This study	RCT	China	6.73–14.49	105	1	NIDEK OPD-Scan III	A, 0.01%	11.05 (1.64)	0.88 (0.68)	1.40 (0.92)
							Placebo	10.75 (1.79)	0.08 (0.72)	0.88 (0.77)
Pérez-Flores et al. (2021) [13]	An observational study	Spain	6–12	92	1	IOL Master, Zeiss	A, 0.01%	9.76(1.93)	0.74(1.26)	
Hieda et al. (2021) [32]	RCT	Japan	6–12	171	1	An infrared camera	A, 0.01%	8.99 (1.44)	0.26 (0.83)	0.09 (0.71)
							Placebo	8.98 (1.50)	0.13 (0.85)	0.14 (0.72)
Saxena et al. (2021) [33]	RCT	India	6–14	100	1	PLR-200TM monocular infrared pupillometer	A, 0.01%	10.6 (2.2)	0.02 (0.47)	0.05 (0.43)
							Placebo	10.8 (2.2)	− 0.06 (0.58)	− 0.12 (0.64)
Fu et al. (2020) [19]	RCT	China	6–14	400	1	NIDEK, AR-1, Japan	A, 0.02%	9.4 (1.8)	0.79	
							A, 0.01%	9.3 (1.9)	0.7	
							Placebo	9.5 (1.4)	0.12	
Chia et al. (2012) [9]	RCT	China, Taiwan	6–12	400	1	Procyon 3000 pupillometer; Neuroptics pupillometer	A, 0.5%	9.7 (1.5)	3.11 (1.08)	3.50 (1.05)
							A, 0.1%	9.7 (1.6)	2.42 (0.91)	2.77 (1.03)
							A, 0.01%	9.5 (1.5)	0.91 (0.78)	1.15 (0.78)
Yam et al. (2019) [10]	RCT	China, Hong Kong	4–12	438	1	NIDEK OPD-Scan III	A, 0.05%	8.45 (1.81)	1.03 (1.02)	0.58 (0.63)
							A,0.025%	8.54 (1.71)	0.76 (0.90)	0.43 (0.61)
							A, 0.01%	8.23 (1.83)	0.49 (0.80)	0.23 (0.46)
							Placebo	8.42 (1.72)	0.13 (1.07)	0.02 (0.55)

The Atropine Treatment of Myopia trials (ATOM 2) phase 2 study assessed changes in pupil size in eyes treated with 0.01%, 0.1%, and 0.5% atropine after cessation [34]. After cessation of atropine, mesopic and photopic pupil sizes in all groups reduced continuously in the following 12 months. Eight months after cessation, the pupil sizes were slightly smaller than in the first screening visit in all three groups [34]. Zhu et al. also reported that pupil size returned to pre-atropine levels at the end of follow-up in Chinese children [35]. Our study also showed a recovery of pupil metrics after the cessation of treatment. Thus, adverse effects of atropine could be eliminated by a gradual cessation and elimination of atropine.

Some studies indicated that the change of PD after atropine treatment may contribute to myopic progression. Fu et al. reported that the AL of children with a smaller PD might increase rapidly while receiving atropine treatment, indicating that changes in PD may suggest the response to the effects of low-concentration atropine [36]. It also suggested that girls with slower myopia progression reported more photophobia issues than girls with a higher progression rate [37]. Consistent conclusions have been made in orthokeratology lens treatment trials. Larger pupil diameters facilitated the effect of the orthokeratology lens to slow axial growth in myopia [21, 38].

Some mechanisms have been postulated to explain the association between myopia and pupil metrics. Pupil size could determine the amount of light entering the eyes, and larger pupils could allow more light to reach the peripheral retina, resulting in more peripheral defocus and thus affecting myopic progression [21, 39]. Wong et al. found that PD after 20 min of dark adaptation in the early-onset myopes was 4.52 mm, which was significantly smaller than that in the emmetropes (5.21 mm) (*P* < 0.05) [40]. They speculated that small PD may play an essential role in the pathogenesis of early-onset myopia via its effect on the depth of focus [40]. However, our study did not find the impact of pupillary parameters on the myopic progression of 0.01% atropine, which may be due to the small sample size and short period. Further research is still needed to illustrate whether the pupil size of participants could affect myopia control efficacy among interventions.

### Strength and limitation

This study was based on a randomized, double-blind trial and continued following up on pupil metrics during and after atropine treatment. There are some limitations of the present study. First, an acknowledged weakness of the study was the lack of baseline measurement of pupil metrics for the atropine-placebo group before atropine treatment. We planned to measure the pupil parameters of the subjects in the initial protocol. Unfortunately, since the measuring instrument needed to be purchased from abroad, the study had been conducted for 1 year when the instrument arrived at the experimental site. Regrettably, we failed to measure the pupil metrics at the beginning of the first year. Since the placebo-atropine group served as a blank control in the first year, the pupil was not affected by the drug, we used the pupil metrics of the placebo-atropine group before atropine treatment as a comparison group. These two groups were matched for baseline characteristics, so the direct comparison between the two groups was deemed appropriate. Second, the dropout rate was high because of the pandemic sparked by the Covid-19, whereas there was no statistical difference between lost-to-follow and continuing participants. More extensive studies with different atropine concentrations and longer follow-ups may help validate the specific long-term effects of atropine on pupil metrics.
